# Sox2-CreER mice are useful for fate mapping of mature, but not neonatal, cochlear supporting cells in hair cell regeneration studies

**DOI:** 10.1038/srep11621

**Published:** 2015-06-25

**Authors:** Bradley J. Walters, Tetsuji Yamashita, Jian Zuo

**Affiliations:** 1 Dept. of Developmental Neurobiology, St. Jude Children’s Research Hospital, Memphis, TN 38105, USA

## Abstract

Studies of hair cell regeneration in the postnatal cochlea rely on fate mapping of supporting cells. Here we characterized a Sox2-CreER knock-in mouse line with two independent reporter mouse strains at neonatal and mature ages. Regardless of induction age, reporter expression was robust, with CreER activity being readily detectable in >85% of supporting cells within the organ of Corti. When induced at postnatal day (P) 28, Sox2-CreER activity was exclusive to supporting cells demonstrating its utility for fate mapping studies beyond this age. However, when induced at P1, Sox2-CreER activity was also detected in >50% of cochlear hair cells, suggesting that Sox2-CreER may not be useful to fate map a supporting cell origin of regenerated hair cells if induced at neonatal ages. Given that this model is currently in use by several investigators for fate mapping purposes, and may be adopted by others in the future, our finding that current protocols are effective for restricting CreER activity to supporting cells at mature but not neonatal ages is both significant and timely.

Mammals have limited capacity to regenerate cochlear hair cells after ototoxic damage^1^,^2^,^3^,^4^,^5^,^6^,^7^, whereas non-mammalian vertebrates can spontaneously regenerate damaged hair cells from surrounding supporting cells by either direct transdifferentiation or mitotic regeneration^8^,^9^,^10^,^11^,^12^,^13^,^14^. Various genetic and therapeutic approaches have been taken to regenerate hair cells in the postnatal mammalian cochlea. However, in studies where experimental animals appear to possess greater numbers of hair cells than similarly damaged controls, it is often difficult to discriminate between the generation of new hair cells from underlying supporting cells versus the preservation of existing hair cells via intra-cellular repair and/or pro-survival mechanisms^15^,^16^,^17^,^18^,^19^,^20^,^21^,^22^. Indeed, in many studies where induced regeneration has been attempted in mammals, the numbers of hair cells are often less than that of an undamaged cochlea, and any functional benefit often falls dramatically short of normal hearing^1^,^17^,^18^,^23^,^24^,^25^,^26^,^27^, suggesting that a mitigation of hair cell loss and functional recovery of surviving cells is as plausible an explanation as regeneration. Furthermore, several studies demonstrate that a single manipulation (e.g. the addition of a trophic factor or the expression of Atoh1) can act to promote either the phenotypic conversion of supporting cells to hair cell-like cells or the survival of residual hair cells, thus directly illustrating the difficulty in parsing these two putative mechanisms^19^,^21^,^28^,^29^,^30^,^31^. Therefore, it has become critical to demonstrate that newly regenerated hair cells are derived from supporting cells as in non-mammalian vertebrates. To accomplish this traditionally difficult task, investigators are now relying on genetic fate mapping methods where supporting cells specifically are transiently induced to express a permanent marker prior to hair cell damage, so that only subsequently regenerated hair cells derived from supporting cells would also express this marker. Such fate mapping is now commonly performed using CreER-mediated reporter expression in transgenic mice where transient tamoxifen administration allows CreER molecules to enter the nucleus and excise premature stop codons thus enabling permanent expression of a reporter. By utilizing a CreER transgene that possesses a supporting cell-specific promoter, or by knocking the CreER transgene into the locus of a gene that is normally only expressed in supporting cells, permanent reporter expression can be acutely induced and retained only in cells that were supporting cells at the time of tamoxifen administration. We and others have previously demonstrated several mouse lines that are useful for this purpose (Prox1-CreER, Fgfr3-iCreER, Plp-CreER, and Glast-CreER)^5^,^26^,^27^,^32^,^33^; however, these reporters tend to be limited in that they do not allow for fate mapping of all of the supporting cells, but rather are only specific to subsets of supporting cell types.

The Sox2-CreER knock-in mouse line represents a promising model for the fate mapping of supporting cells since Sox2 is expressed in all of the supporting cells, not just certain supporting cell subtypes^2^,^25^,^32^,^34^,^35^,^36^. However, several lines of evidence suggest the presence of Sox2 expression / transcriptional activity in cochlear hair cells at postnatal and adult ages^2^,^27^,^37^,^38^,^39^,^40^,^41^. It is therefore critical to validate at what age, if any, that tamoxifen induction of this Sox2-CreER line will label only supporting cells and not hair cells. Finding an appropriate induction age is critical since the induction of reporter expression in any existing hair cells at the time of tamoxifen induction would defeat the purpose of such fate mapping and could not then be effectively used to support the conclusion that newly regenerated hair cells are derived from supporting cells in hair cell regeneration studies. Additionally, the vast majority of regeneration studies in the mouse cochlea are undertaken at neonatal ages due to greater plasticity of the cochlea and better survival of the tissue in culture at younger ages^1^,^2^,^5^,^6^,^20^,^26^,^27^,^36^,^42^,^43^,^44^,^45^,^46^,^47^,^48^,^49^,^50^,^51^.

Here we characterized the Sox2-CreER line at two ages, P1 and P28, by using two independent reporter mouse lines (Ai14 and mT/mG). Ai14 is a highly sensitive Cre reporter line where the tdTomato reporter gene, following Cre mediated excision of the loxP-Stop-loxP cassette, is driven by the Rosa26-CMV β-actin enhancer (CAG) promoter^5^,^32^,^33^,^52^,^53^. The mT/mG line is also driven by the Rosa26-CAG promoter, which is then followed by a loxP-mtdTomato-Stop-loxP–mGFP cassette. In the mT/mG mice, Cre negative cells, or uninduced Cre-positive cells, constitutively express mtdTomato, but upon Cre or CreER mediated excision of the mtdTomato, mGFP instead is expressed^54^. In this latter model, both mtdTomato and mGFP are membrane bound, while tdTomato in the Ai14 reporter line is mainly cytoplasmic and nuclear.

To test the supporting cell specificity of the Sox2-CreER line, we induced Sox2-CreER; Ai14 mice via two intraperitoneal tamoxifen injections (75 mg/kg body weight) spaced 24 hours apart between P0-P1, and analyzed the cochleae at P8 (Fig. 1A,B). Immunostaining of myosin-VIIa (Myo7a) and Sox2 allowed us to label hair cells and supporting cells, respectively, while the fluorescent reporter (tdTomato) was visualized directly using fluorescent, laser scanning confocal microscope. We quantified cell numbers of each supporting cell subtype and inner or outer hair cell subtype based on cell locations and immunolocalization of Myo7a and Sox2 in these architecturally organized organs of Corti. As expected, we found that a vast majority (>85%) of supporting cells are double positive for the Cre-activated reporter (tdTomato) and Sox2 (Fig. 1A,B); however, we also found that ~50% of Myo7a positive hair cells were also labeled by the Cre-activated tdTomato reporter (Fig. 1A,B). This was most noticeable in the apical turns of the cochleae, where 83.92 ± 10.32% of Myo7a positive hair cells were also tdTomato positive, as compared to 36.15 ± 19.41% in the middle turns, and 19.78 ± 16.47% in the basal turns.

Similarly, we analyzed the cochleae of Sox2-CreER; mT/mG mice at P38 (Fig. 2) after tamoxifen (75 mg/kg body weight) had been administered by a single intraperitoneal injection at P1^25^. In the apical turns, we found that only ~40% of the Myo7a positive hair cells remained mtdTomato positive, whereas nearly 60% of the Myo7a positive hair cells were mGFP positive, suggesting Sox2-CreER activity. These results are consistent with what we found in Sox2-CreER; Ai14 mice (Fig. 1A,B) and suggest that neonatal tamoxifen induction in the Sox2-CreER line may not be a suitable approach for fate mapping cochlear supporting cells.

In contrast, we also injected Sox2-CreER; Ai14 mice with tamoxifen (250 mg/kg body weight) by a single injection at P28, and analyzed the cochleae at P35. Under these conditions, we found that many (>85%) Sox2 positive supporting cells, but no hair cells (immunostained for Myo7a), were labeled with the Cre-activated reporter (tdTomato) (Fig. 1C,D).

Our studies demonstrate that the Sox2-CreER line can be used as an effective genetic tool for the fate mapping of cochlear supporting cells following mature, but not neonatal tamoxifen induction. In support, Sox2 has been demonstrated to be expressed in progenitor cells, developing hair cells and supporting cells in late mouse embryos^35^,^38^,^39^,^55^,^56^,^57^, and while it is clearly down-regulated to some extent in differentiating hair cells between E16.5 and P4, several reports suggest that Sox2 is still expressed in cochlear hair cells at postnatal ages^2^,^27^,^37^,^38^,^39^,^40^,^41^. Our results here, using two independent fate mapping reporters, strongly support the hypothesis that Sox2 is indeed expressed in cochlear hair cells at P0-P1. This is of significance since several previously published studies have suggested the use of the Sox2-CreER line to fate map presumptive supporting cells following P1 induction^2^,^25^,^36^. While one of these reports has recently been corrected to clarify that the induction was carried out at P21 rather than P1^25^, it is likely that investigators may still seek to use this mouse line to lineage trace supporting cells. The data presented here, which are corroborated by a previous report using the mT/mG reporter^2^, contradict that notion, suggesting limited or no utility for fate mapping since such a large number of cochlear hair cells exhibit Cre activity in response to neonatal induction of Sox2-CreER. However, this does not completely negate the usefulness of the Sox2-CreER mouse line for the fate mapping of supporting cells as the data also show that the Sox2-CreER line is specific to, and robustly expressed in, cochlear supporting cells following induction at later (i.e. young adult) ages.

## Methods

### Animals

Mice were housed under a 12 h light/dark cycle with free access to food and water. The Animal Care and Use Committees of St. Jude Children’s Research Hospital approved all of the protocols performed in this study and the methods were carried out in accordance with the approved guidelines. Sox2-CreER mice were generously provided by Konrad Hochedlinger, Harvard University, and are now available from Jackson Laboratory (Stock #017593). The mT/mG (Stock# 007576) mice were purchased from The Jackson Laboratory. Genotyping of Sox2-CreER and mT/mG mice were previously described^34^,^54^.

### Histological analysis

In order to look at mGFP and mtdTomato distributions in organ of Corti, the Sox2-CreER; mT/mG mice were anesthetized by intraperitoneal injection with Avertin (0.5 mg/kg body weight) and fixed transcardially using fixative solution (4% paraformaldehyde in 0.1 M phosphate buffer (pH 7.4)). The inner ears were perfused via round window and fixed using the fixative solution at room temperature for 4 hours. After the fixation, the cochleae were carefully dissected out. Primary antibodies used were rabbit anti-myo7a (Proteus Bioscience, at 1:100), goat anti-Sox2 (Santa Cruz, at 1:500). The immunofluorescence was visualized by adding Alexa Fluor 405 goat anti-rabbit IgG (H + L), or Alexa Fluor 488 donkey anti-goat IgG (H + L) and Alexa Fluor 647 donkey anti-rabbit IgG (H + L) (Molecular Probes, Eugene, OR, USA). Fluorescence images were taken from N = 3 Sox2-CreER-Ai14 mice at P8 (following P0-P1 induction), N = 4 Sox2-CreER;Ai14 mice at P35 (following P28 induction), and N = 3 Sox2-CreER;mT/mG mice at P38 (following P1 induction). Images were acquired with a Zeiss Axiophot2 microscope equipped with a 40× oil immersion and 1.4 NA objective using a LSM700 confocal laser scanning image system (Carl Zeiss, Jena, Germany). For the Sox2-CreER;Ai14 mice, hair cells and supporting cells were counted from six representative images per cochlea, two from each turn (apical, middle, and basal). For the Sox2-CreER;mT/mG mice, cells were counted from two representative images per cochlea, both in the apical turn.

## Additional Information

**How to cite this article**: Walters, B. J. *et al.* Sox2-CreER mice are useful for fate mapping of mature, but not neonatal, cochlear supporting cells in hair cell regeneration studies. *Sci. Rep.* **5**, 11621; doi: 10.1038/srep11621 (2015).

## Figures and Tables

**Figure 1:**
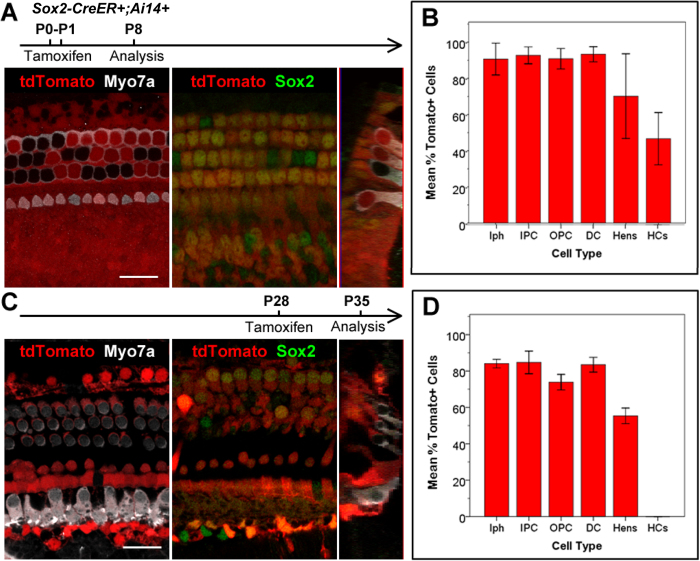
A,B. Sox2-CreER labels >85% supporting cells and >50% inner and outer hair cells in the organ of Corti when induced at P0-1. **C**,**D**. Sox2CreER labels only supporting cells in the organ of Corti when induced at P28. Wholemount cochlear confocal images (**A**,**C**) and quantification (**B**,**D**) of Sox2-CreER; ROSA-CAG-Stop-floxed-tdTomato (Ai14) when induced at P0-P1 and analyzed at P8, or induced at P28 and analyzed at P35. Hair cells are labeled with Myo7a antibody (white) in the P0-P1 induced samples (**A**), and in the P28 induced samples (**C**). Sox2 immunostaining (green) labels supporting cells, and tdTomato (red) labels Cre activity. (**B** and **D)**. Percentages of tdTomato+ cells in each subtype of supporting cells (Inner phallengeal cells [Iph], Inner pillar cells [IPC], outer pillar cells [OPC], Deiters’ cells [DC], Hensen cells [Hens]) and hair cells (HCs). Scale bars = 20 μm. Error bars = ±1 standard error in three independent mice.

**Figure 2:**
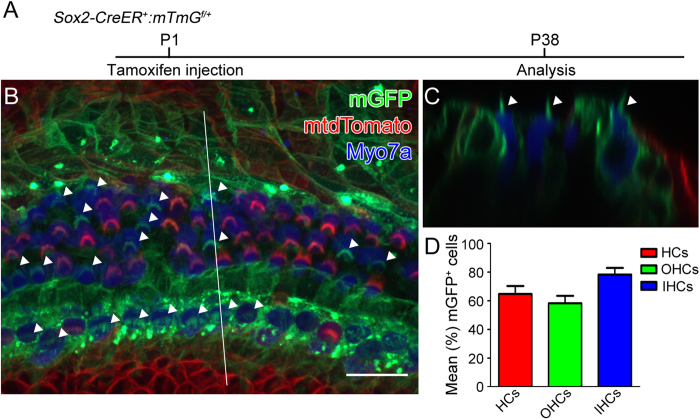
Sox2-CreER labels both supporting cells and hair cells when induced at P1. Tamoxifen was intraperitoneally injected into Sox2-CreER; mTmG mice at P1 and cochleae were analyzed at P38. **A**. The whole mount of apical cochlear turn with mGFP (green) and mtdTomato (red) fluorescence and Myo7a immunostaining (blue) using LSM700 confocal laser scanning microscope. **B**. The optical xz-plane of the line in **A**. Arrowheads label mGFP^+^ hair bundles. Scale = 20 μm. **C**. Percentages of mGFP^+^ hair cells in a 300-μm region from the apical turn. Error bars = ±1 standard error, N = 3 independent mice.
